# Visual Confirmation of Residual Erythrocytes and Platelets in Human Plasma

**DOI:** 10.1177/11772719261481222

**Published:** 2026-09-21

**Authors:** Mendel Engelaer, Rienk Nieuwland, Edwin van der Pol, Jillian W. P. Bracht

**Affiliations:** 1 Department of Biomedical Engineering and Physics, 571151 Amsterdam UMC, Amsterdam, Netherlands; 2 Cancer Center Amsterdam, Imaging and Biomarkers, 26066 Amsterdam UMC, Location University of Amsterdam, Amsterdam, The Netherlands; 3 Amsterdam Cardiovascular Sciences, Atherosclerosis and Ischemic Syndromes, 26066 Amsterdam UMC, Location University of Amsterdam, Amsterdam, The Netherlands; 4 Laboratory of Experimental Clinical Chemistry, 26066 Amsterdam UMC, Amsterdam, Netherlands

**Keywords:** biomarker, plasma preparation, platelets, erythrocytes, biobanks, sample quality, flow cytometry, sorting, microscopy

## Abstract

**Background:**

Human plasma stored in biobanks serves as a starting material for many biomarker studies. Despite being termed “platelet-free plasma”, plasma still contains erythrocytes and platelets at concentrations below the limit of detection of routine haematology analysers, approximately 10^6^ mL^-1^. The presence of these residual cells affects biomarker studies of extracellular vesicles (EVs). In previous work, we used flow cytometry to study residual cells in double-centrifuged plasma. However, because direct visualization of such low cell concentrations is technologically challenging, residual cells detected by flow cytometry in plasma have not yet been directly visualized.

**Objectives:**

Our goal is to visualize residual erythrocytes and platelets in double-centrifuged plasma and confirm that the flow cytometry events we previously detected indeed correspond to these cell types.

**Design & Methods:**

We developed a workflow combining cell sorting, flow cytometry, and super-resolution microscopy to enrich and image residual erythrocytes and platelets, and applied it to the plasma of a single donor.

**Results:**

Flow cytometric analysis from the plasma of a healthy donor revealed concentrations of 4.5×10^5^ mL^-1^ for erythrocytes and 3.05×10^5^ mL^-1^ for platelets, consistent with previous reports. Sorting enriched these cells by 64-fold and 40-fold, respectively, enabling super-resolution microscopic imaging. Microscopy confirmed the presence of activated erythrocytes expressing CD235a and platelets expressing CD63.

**Conclusions:**

For the first time, we linked flow cytometry measurements of cells in double-centrifuged plasma to super-resolution microscopy images, thereby providing a visual confirmation of the presence of erythrocytes and platelets. The presence of these cells further underscores the need to account for them in EV-focused biomarker exploration.

## Introduction

Biobanks play a critical role in biomarker research, with plasma being the most commonly stored and used body fluid for the discovery and validation of new biomarkers.^1-4^ Plasma quality is particularly important in studies on subcellular particles such as extracellular vesicles (EVs). EVs are membrane-bound particles released by cells that carry molecular cargo and are increasingly studied for their diagnostic and therapeutic potential. However, plasma may contain contaminants such as hemolysis products, residual cells, and lipoproteins, which can affect downstream analyses of EVs.^5^

In EV research, plasma is often prepared using a protocol that removes cells from anticoagulated blood by centrifuging twice at 2.500 *g* for 15 minutes.^6-8^ The resulting plasma was commonly referred to as “platelet-free plasma”. However, this term is misleading, as such plasma still contains residual cells, primarily platelets and erythrocytes, including erythrocyte ghosts (hemoglobin-depleted erythrocytes), as first observed by cryo-transmission electron microscopy in 2014.^9^ More recently, we used flow cytometry to show that the concentration of residual platelets in double-centrifuged plasma ranges from 1×10^5^ to 1×10^8^ mL^-1^.^10^

Based on the measured concentrations of residual platelets, we estimated that the volume fraction of residual platelets is in the same order of magnitude as that of EVs.^10^ Residual cells, particularly platelets, overlap with EVs in physical properties such as density and biochemical composition, and are hard to separate from EVs.^10-13^ Consequently, the presence of residual cells can significantly confound results from studies in which, for example, the concentration and/or molecular cargo of EVs is analyzed.^10,12^ Since most biomarker studies focusing on EVs do not reach the clinic,^14,15^ reducing confounding factors such as residual cells is important to increase sample quality and thereby increase reproducibility and comparability of EV biomarker studies.^16^

Despite these findings, the presence and impact of residual cells in plasma have thus far largely been ignored and/or overlooked for three reasons. First, the concentration of residual cells in plasma is below the detection limit of routine hematology analyzers.^12^ Hence, detection of residual cells requires instruments capable of analyzing larger volumes than hematology analyzers, such as flow cytometers. Second, the impact of residual cells on biomarker assays, other than platelet-EV concentrations and EV-miRNA,^10,12^ has not been systematically investigated and remains unknown. Third, a flow cytometer detects fluorescent and light scattering from residual cells, but confirmation by an independent method that these particles are indeed cells is still lacking. So far, visual confirmation has been reported only once,^9^ but not after sorting, leaving the possibility that the detected particles by flow cytometry may also be large EVs or cell fragments.^10^

Because “seeing is believing,” the aim of this manuscript is to provide visual confirmation of residual erythrocytes and platelets in “platelet-free plasma”. To visualize these rare cells, we developed a workflow using a microchip-based valve-controlled sorter to enrich cells, a flow cytometer to quantify cells, and a super-resolution microscope to image the enriched cells. With this workflow, we provide direct visual confirmation that the flow cytometry signals we previously detected indeed correspond to residual erythrocytes and platelets.

## Material and Methods

### Plasma Preparation

Blood was collected from a healthy female donor (age 29) on three different days as technical replicates. The donor denied having a disease or using drugs and/or medication. This manuscript presents results from one of three representative platelet experiments and one erythrocyte experiment. Venous blood was collected using a 21-Gauge needle without a tourniquet. The first 2-3 mL of blood was discarded to avoid that activation of endothelial cells and platelets during vein puncture would affect the measurement results. Two tubes of ethylene diamine tetra acetic acid (EDTA) blood (6 mL; BD Biosciences) were then collected from the donor. Blood was processed within 15 minutes after withdrawal.

Plasma was prepared by centrifugation of whole blood, according to the guidelines of the American Heart Association.^8^ In short, whole blood was centrifuged for 15 minutes at 2,500 x g and 20°C, with the centrifuge acceleration set at 9, and deceleration set at 1, using a Rotina 380 R centrifuge with a swing-out rotor and a radius of 155 mm (Hettich Zentrifugen, Tuttlingen, Germany). Single centrifuged plasma was collected to exactly 10 mm above the buffy coat, and 3.5 mL plasma was then transferred to a 15 mL polypropylene tube (Greiner Bio-One B.V., Alphen aan den Rijn, The Netherlands). The plasma was then centrifuged for 15 minutes at 2,500 x g and 20°C, with the centrifuge acceleration set at 9, and deceleration set at 1. Double centrifuged plasma was collected to exactly 10 mm above the cell pellet.

The concentration of residual cells in double centrifuged plasma was directly assessed by flow cytometry (Cytek Northern Lights, Cytek Biosciences, Seattle, USA) and this plasma was used as the input material for sorting experiments on the same day. Remaining plasma was aliquoted into 1.5 mL low-protein binding Eppendorfs (Thermo Fisher Scientific, US) and stored at -80˚C.

### Immunofluorescence Staining

In this study, we focus on both erythrocytes and platelets as residual cells in plasma. We do not distinguish between intact erythrocytes and erythrocyte ghosts and refer to both collectively as erythrocytes. Before staining, all samples were diluted 2-fold in Dulbecco’s phosphate buffered saline (DPBS). Antibodies were diluted in DPBS and aggregates were removed by centrifugation at 18,890 g for 5 min at 20 °C. For erythrocyte sorting, 1,400 μL of the sample was stained with 175 μL CD235a-APC (for erythrocytes) and 175 μL CD61-PE (to quantify platelet presence within the erythrocyte-sorted fraction). Stained samples were kept in the dark for 30 minutes at room temperature. After the staining, samples were diluted in 15,480 μL of DPBS to decrease background fluorescence from unbound reagents. For platelet sorting, 1,000 μL of the sample was stained with 125 μL CD61-PE (for platelets), 125 μL CD235a-APC (to assess erythrocyte contamination), and 125 μL CD62p-BV421 (to evaluate platelet activation). After staining, the samples were diluted in 12,105 μL DPBS. An overview of the staining reagents can be found in Supplemental Table 1 of the supplemented MIFlowCyt.

### Sorting of Residual Particles

Erythrocytes and platelets were sorted using a MACSQuant Tyto Cell Sorter (Miltenyi Biotec, Germany). The sorter works with disposable cartridges that contain a microfluidic chip, where particles are sorted using a valve. The sample is pressurized, and no sheath fluid is used, minimizing the dilution of the particles during sorting. The sorter is equipped with a 405-nm, 488-nm, and 640-nm laser. 12.5 mL of CD235a-APC erythrocytes were sorted using the backscatter-violet and APC channel. 13 mL of CD61-PE labeled platelets were sorted using a sorting gate on the backscatter-violet and PE channel. Sort gates were set based on 1x2500 g centrifuged cell-rich plasma. Detector gains were set as follows: BSV 610 V, BSB 700 V, BSR 640 V, B2 470 V, and R1 630 V. High-speed cartridges were used with an average flow rate of 8 mL/hour and a maximum input volume of 10 mL. To measure more than 10 mL, the cartridges were reprimed after measuring 10 mL, after which the remaining volume was measured. A mixer in the input chamber mixed the sample during acquisition at a rate of 800 rpm. The temperature was set to room temperature. The sorted material was extracted for flow cytometry and microscopy measurements. Remaining post-sorting material was aliquoted into 1.5 mL low-protein binding Eppendorfs (Thermo Fisher Scientific, US) and stored at -80˚C. Sorting experiment details, including measurement times, spectral filters, trigger and threshold settings, fluorescence and scattering calibration, and sort gate descriptions, are reported in the supplemented MIFlowCyt.^17^

### Flow Cytometry

A calibrated Northern Lights flow cytometer (Cytek, USA) was used to measure the concentration of erythrocytes and platelets in labeled, unlabeled, sorted, and unsorted samples. The flow cytometer is equipped with a 405 nm, 488 nm and 640 nm wavelength laser. Light scattering calibrations were performed using Rosetta Calibration (v2.05, Exometry, The Netherlands). The trigger threshold was set on the side scattering detector at 750 arbitrary units (a.u.), corresponding to a side scattering cross section for erythrocytes of 337 nm^2^ and 341 nm^2^ for platelets. The SSC-A parameter was used for side scattering, B4-H for PE fluorescence and R1-H for APC fluorescence. The gain of the detectors was set at 5 a.u. for the SSC detector, 1,250 a.u. for the B4 detector, and 500 a.u. for the R1 detector. Erythrocytes were selected by gating particles expressing anti-human CD235a-APC with fluorescence intensities between 2.2×10^5^ and 1.1×10^6^ molecules of equivalent soluble fluorophores (MESF) and with a scattering cross-section between 6.7×10^3^ and 2.4×10^4^ nm^2^. Platelets were selected by gating particles expressing anti-human CD61-PE with fluorescence intensities between 4.9×10^3^ and 1.6×10^5^ MESF and with scattering cross-sections between 1.0×10^3^ and 9.7×10^4^ nm^2^. Samples were measured for 240 seconds at a flow rate of 35 μL/min. Flow cytometry experiment details, including spectral filters, fluorescence and scattering calibration, and gating descriptions, are reported in the supplemented MIFlowCyt.^17^

### Super-Resolution Microscopy

Direct stochastic optical reconstruction microscopy (dSTORM) imaging of sorted cells was carried out on a super-resolution Nanoimager S running NimOS version 1.4 (Oxford NanoImaging). The images were acquired using a 100×1.4 NA Olympus objective, sCMOS camera (Hamamatsu orca flash 4.0 V3). Capture and staining procedures were performed according to manufacturer protocol. In short, erythrocytes and platelets were captured on ONI human EV Profiler kit v2.0 cartridges, using CD235a-biotin (Miltenyi Biotec) or CD63-biotin (EVP2, ONI) for erythrocytes and platelets, respectively. After capture, erythrocytes and platelets were labeled with a lipophilic membrane marker (panEV dye, EVP2, ONI) and either CD235a-AF647 (Miltenyi Biotec, erythrocytes) or CD63-CF583R (EVP2, ONI, platelets). In case of CD235a-AF647, a final concentration of 70 nM was used for capture.

Single molecule data acquisition on the Nanoimager was performed in the NimOS Light program in total internal reflection fluorescence illumination (TIRF) mode. The following parameters were used during acquisition: 640 nm laser set to 75% laser power, the 560 nm laser at 40% laser power, and the 488 nm laser set to 75% laser power. The number of frames to be recorded for the 640 and 560 nm lasers was set to 1,000. For the 488 nm laser the number of frames to be recorded was set to 3,000. Images were further analyzed in ONI’s cloud-based data analysis platform (CODI).

### Fluorescence Microscopy

Fluorescence microscopy images of the sorted CD235a-APC labeled erythrocytes and the CD61-PE labeled platelet samples were made. 5 μL of sample was loaded on a 76 × 26 mm glass microscope slide and covered with a #1 18x18 mm glass coverslip (Menzel-Glaser, Germany). Images were made on a Nikon ECLIPSE E600 microscope (Nikon, Japan) with a mercury light source (Nikon, Japan). A G-2A filter set containing a 510-560 nm excitation filter, a 576 nm dichroic mirror, and a 590 nm barrier filter was used together with a Nikon 40x/0.75 NA Plan Fluor objective. Images were taken with a Nikon DS-Fi2 camera (Nikon, Japan), and the exposure time was set at 1 second. The pixel calibration was 0.12 μm/px, measured using a stage micrometer.

## Results

### Cell Sorting and Flow Cytometry

We first quantified the concentrations of residual erythrocytes and platelets in the double-centrifuged plasma using flow cytometry. The flow cytometry measurements in Figure 1A and B depict erythrocytes and platelets (red gates) as clearly distinct populations before sorting. Erythrocyte and platelet concentrations in double-centrifuged plasma were 4.52×10^5^ mL^-1 ^and 3.05×10^5^ mL^-1^, respectively, consistent with previous reports.^10,12^ Two additional independent cell concentration measurements have been reported in Supplemental Figure 1. Next, we sorted erythrocytes and platelets. The enrichment of both cell types is shown in Figure 1C and D. Compared to the initial measurements, the erythrocyte concentration increased 64-fold, and the platelet concentration increased 40-fold.

**Figure 1.**
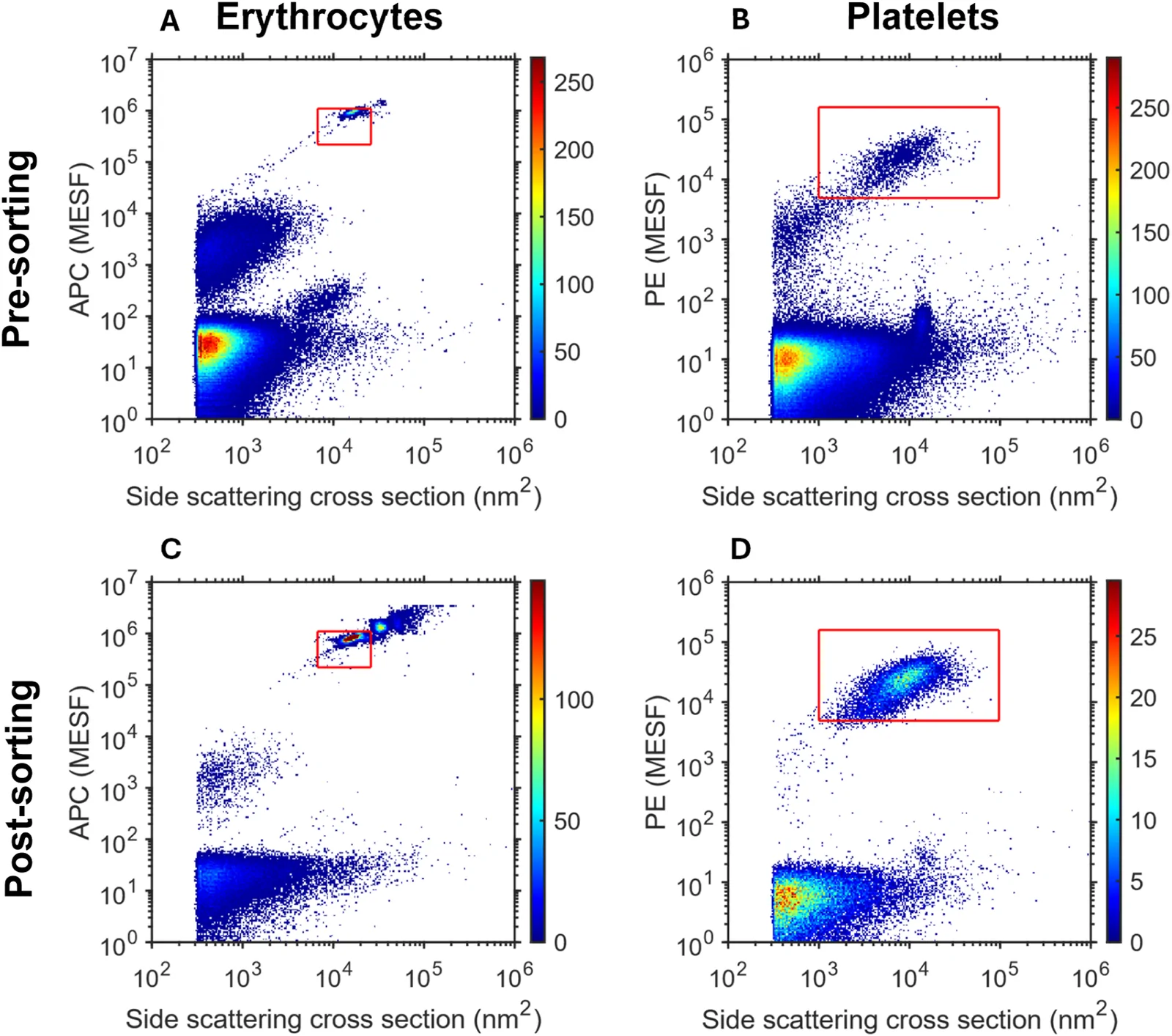
Flow cytometry of erythrocytes (CD235a-APC, left column) and platelets (CD61-PE, right column). (A) Erythrocytes in double-centrifuged plasma before sorting: red gate with 3,062 particles (4.52×10^5^ mL^-1^). (B) Platelets in double-centrifuged plasma before sorting: red gate with 2,050 particles (3.05×10^5^ mL^-1^). (C) Sorted erythrocytes: red gate with 19,749 particles (2.89×10^7^ mL^-1^). (D) Sorted platelets: red gate with 8,383 particles (1.23×10^7^ mL^-1^). In A and C, the gate was positioned to exclude populations with MESF and scattering cross-section values that are likely aggregates.

### Super-Resolution and Fluorescence Microscopy

To confirm the presence of residual erythrocytes and platelets in the sorted material, we imaged the sort fractions using super-resolution imaging (dSTORM, ONI) and fluorescence microscopy. Figure 2A and B show super-resolution images of the sorted fractions, confirming the presence of erythrocytes and platelets. The platelets are activated, indicated by the protruding pseudopods, expressing CD63. The erythrocytes are visible as oval particles expressing CD235a. In addition, Figure 2C shows fluorescence microscopy images of clumped erythrocytes, confirming a previously described morphology.^18^

**Figure 2.**
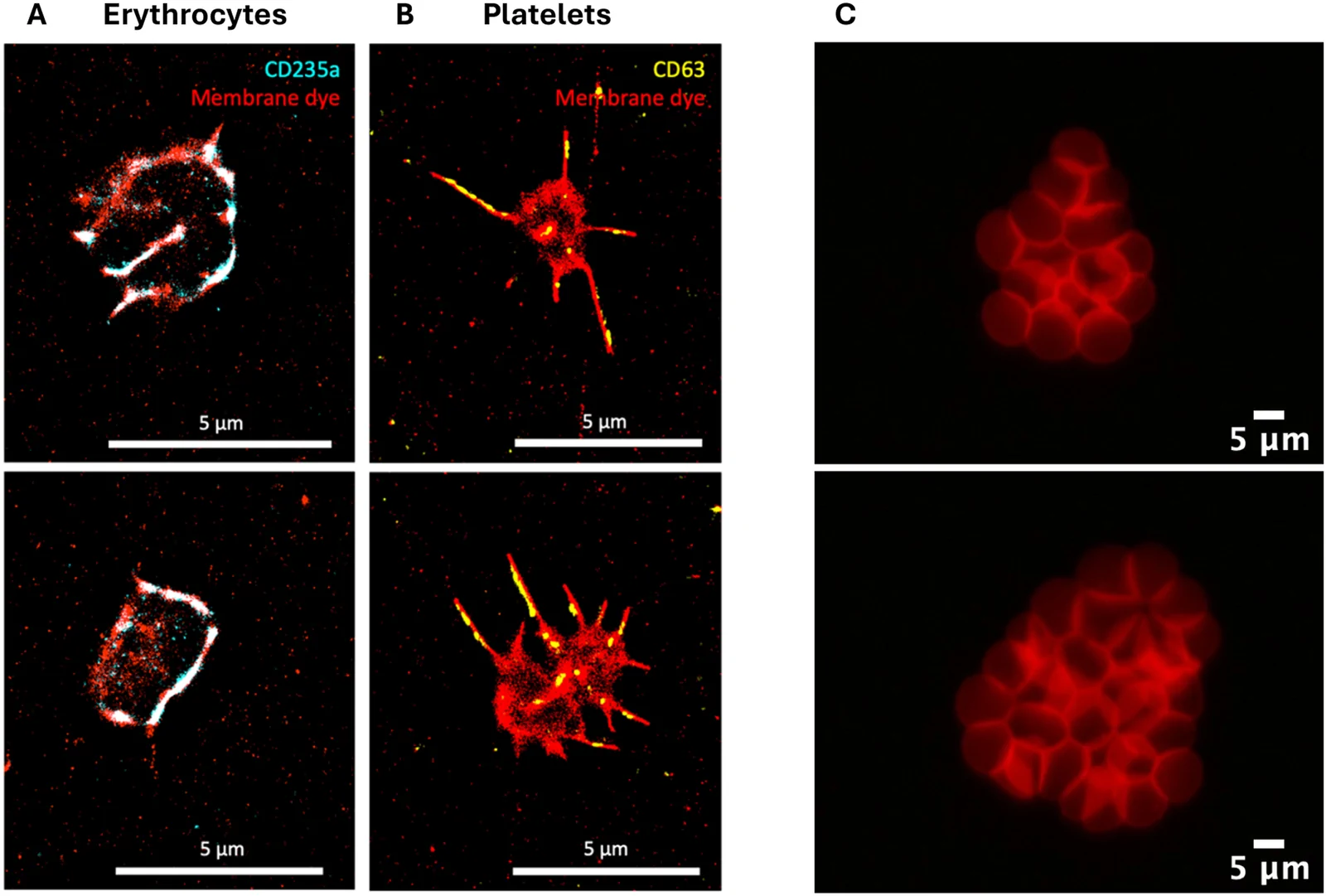
(A) Two dSTORM images of erythrocytes in the sorted sample captured with CD235a-biotin and labeled with CD235a-AF647 (blue) and membrane dye (red). (B) Two dSTORM images of activated platelets in the sorted sample captured with CD63-biotin and labeled with CD63-CF583R (yellow) and membrane dye (red). (C) Fluorescence microscopy images of aggregated erythrocytes (CD235a).

## Discussion

The presence of confounding factors, such as platelets and erythrocytes, can affect biomarker research focusing on EVs.^12,15,16^ Several flow cytometry studies have reported particles in plasma that are larger and more fluorescent than typical EVs.^10,12^ However, these studies did not provide independent visual evidence that these particles are indeed residual blood platelets or erythrocytes. The lack of independent evidence to confirm the identity of the measured particles has raised questions about whether these particles are residual blood cells, EVs, or cell fragments. Identifying these residual cells or cell fragments is important because residual cells can compromise the composition of (biobanked) plasma samples and potentially influence the outcomes of biomarker measurements.^12^ In this study, we present independent evidence confirming that double-centrifuged plasma, prepared using a popular protocol for biobanking^6,8^ in the research field of EVs, contains residual platelets and erythrocytes.

By first enriching the residual cells using a cell sorter, we were able to visually confirm the presence of residual cells in the double-centrifuged plasma using fluorescence and super-resolution microscopy. Super-resolution microscopy allowed us to clearly resolve CD63-positive pseudopods on activated platelets, which was not possible in the earlier cryo-EM imaging of residual platelets by Arraud et al (2014). Additionally, by sorting these cells, we developed a workflow that enables further research into the 1) characterization of the physical features of the residual erythrocytes and platelets, such as investigating why these cells are not removed by centrifugation; and 2) characterization of the biochemical composition of the residual erythrocytes and platelets to reveal their possible impact on downstream biomarker assays.

Besides characterizing the residual cells, manufacturers should focus on investigating, developing, and applying new technologies to remove *all* cells from blood when preparing plasma. For example, Bettin et al 2022 and Bracht et al 2023 showed that 800-nm track-etched polycarbonate filters effectively remove platelets and erythrocytes from plasma. A disposable variant of these filters would provide an affordable, effective, and clinically applicable procedure to achieve true platelet-free plasma. In addition, there are other emerging technologies that may outperform centrifugation regarding cell removal and could potentially replace centrifugation in the future, such as microfluidics or acoustic sorting.^19,20^

Until new solutions for cell removal are developed and implemented, the presence of residual erythrocytes and platelets in plasma is difficult to overcome. Therefore, it is recommended to report the concentration of residual cells in plasma.^5,13^ Reporting residual cell concentrations is expected to improve the consistency and comparability of results across studies.

### Limitations

The main limitation of this study is that the sorting and imaging experiments were performed on the plasma of a single donor. However, we expect our visual confirmation that the flow cytometry signals in double-centrifuged plasma correspond to residual erythrocytes and platelets to be generalizable, since the measured platelet and erythrocyte concentrations are comparable to those previously reported.^10,12^ Another limitation is that with super-resolution microscopy we used the same antibody for both capturing and labeling the erythrocytes and platelets, specifically CD63 and CD235a. Consequently, particles with low epitope numbers could be missed due to antigen saturation. Furthermore, we did not distinguish erythrocytes from erythrocyte ghosts, which are erythrocytes that have been depleted of their hemoglobin.^9^ Sorting these erythrocytes (ghosts) and comparing them to erythrocytes in whole blood using either microscopy or density gradient centrifugation might provide more information to differentiate between the two, which could explain why these residual erythrocytes remain after centrifugation. Similarly, we did not quantify the number of EVs, aggregates, or cell fragments present in the sorted fractions. Consequently, the purity of the erythrocytes and platelets relative to other particle populations remains unknown and requires further investigation. Finally, the imaged activated platelets were present in plasma that was collected in EDTA-containing blood collection tubes. EDTA strongly chelates calcium ions and completely prevents platelet activation.^21^ Further investigation is necessary to understand at what stage of the protocol the platelets became activated, since there are a variety of possible explanations for activation, such as shear stress or contact with glass surfaces.

## Conclusion

We provide visual confirmation that the flow cytometry signals observed in double-centrifuged plasma indeed correspond to residual erythrocytes and platelets. These findings highlight an overlooked and underestimated problem and underscore the need for improved technologies and methods to obtain truly cell-free plasma for biobanking.

## Supplemental Material


Supplemental Material - MIFlowCyt Cytek Northern Lights - Visual Confirmation of Residual Erythrocytes and Platelets in Human Plasma
Supplemental Material for Visual Confirmation of Residual Erythrocytes and Platelets in Human Plasma by Mendel Engelaer, Rienk Nieuwland, Edwin van der Pol and Jillian W. P. Bracht in Biomarker Insights.

Supplemental Material - Visual Confirmation of Residual Erythrocytes and Platelets in Human Plasma
Supplemental Material for Visual Confirmation of Residual Erythrocytes and Platelets in Human Plasma by Mendel Engelaer, Rienk Nieuwland, Edwin van der Pol and Jillian W. P. Bracht in Biomarker Insights.

## Data Availability

The datasets generated and/or analyzed during the current study are available in the figshare repository, https://doi.org/10.6084/m9.figshare.c.7667636.^22^

## Appendix

### Abbreviations

APC: Allophycocyanin
BV: Brilliant violet
CD: Cluster of differentiation
DPBS: Dulbecco’s phosphate buffered saline
dSTORM: Direct stochastic optical reconstruction microscopy
EDTA: Ethylene diamine tetra acetic acid
EV: Extracellular vesicle
MESF: Molecules of equivalent soluble fluorophores
miRNA: Micro ribonucleic acid
PE: Phycoerythrin
