# Supplementary material for: Visual Confirmation of Residual Erythrocytes and Platelets in Human Plasma
Source: Biomark Insights. 2026 Sep 21;21:11772719261481222. doi: 10.1177/11772719261481222 (PMC13728427; doi:10.1177/11772719261481222)
Supplement: Supplementary file 1 [file sj-pdf-1-bmi-10.1177_11772719261481222.pdf]

Supplementary Information

MIFlowCyt of study: "Visual confirmation of residual erythrocytes and platelets in human plasma"

This document provides the minimum information required to reproduce the flow cytometry experiments on residual erythrocytes and platelets performed in the study "Visual confirmation of residual erythrocytes and platelets in human plasma". This document is based on a published standardization framework [1].

Contents

1 Experiment overview....2
- 1.1 Contact details....2
  - 1.1.1 Experiment leader....2
  - 1.1.2 Flow cytometry execution....2
- 1.2 Purpose....2
- 1.3 Keywords....2
- 1.4 Experiment variables....2
- 1.5 Experiment design and quality controls....3
- 1.6 Dates....3
- 1.7 Conclusions....3

2 Sample details....3
- 2.1 Sample description....3
  - 2.1.1 Sample source description....3
  - 2.1.2 Sample description....3
- 2.2 Sample collection....3
- 2.3 Sample preparation and storage....3
- 2.4 Sample characteristics....4
- 2.5 Sample dilution....4
- 2.6 Sample staining....4
- 2.7 Fluorescence reagents....5

3 Flow cytometer....6
- 3.1 Model and manufacturer....6
  - 3.1.1 Flow rate and acquisition time....6
  - 3.1.2 Sample mixing and temperature control....6
  - 3.1.3 Light sources....6
  - 3.1.4 Detectors....6
  - 3.1.5 Trigger detector, noise threshold, and sorting channels....7

4 Assay controls....7
- 4.1 Unstained controls....8

5 Data analyses....8
- 5.1 Data sharing....8
- 5.2 Compensation details....8
- 5.3 Calibrations....8
  - 5.3.1 Flow rate....8
  - 5.3.2 Fluorescence calibration....8
  - 5.3.3 Light scattering calibration....9
- 5.4 Sort gate description and boundaries....11

6 References....12

1 Experiment overview

1.1 Contact details

1.1.1 Experiment leader

Name of organization	Amsterdam University Medical Center
Address	Meibergdreef 9<br>PO Box 22660<br>1100 DD<br>Amsterdam<br>The Netherlands
Primary contact name	Mendel Engelaer
Primary contact e-mail address	m.engelaer@amsterdamumc.nl

1.1.2 Flow cytometry execution

Name of organization	Amsterdam University Medical Center
Address	Meibergdreef 9<br>PO Box 22660<br>1100 DD<br>Amsterdam<br>The Netherlands
Contact name	Mendel Engelaer
Contact e-mail address	m.engelaer@amsterdamumc.nl

1.2 Purpose

The goal of this flow cytometry experiment is to determine the concentration of residual erythrocytes and platelets in human blood plasma using a Cytek Northern Lights flow cytometer. Concentrations of erythrocytes and platelets were determined pre- and post-sorting. Sorting was done using a MACSQuant Tyto Cell Sorter (Miltenyi Biotec, Germany). Based on previous results, we hypothesize that the pre-sorting concentration of erythrocytes and platelets in double centrifuged plasma is between $5 \cdot 10^4$ and $2 \cdot 10^6$ mL^{-1} [2]. Through sorting we aim to increase this concentration at least 10-fold to be able to image the erythrocytes and platelets by super-resolution microscopy (dSTORM).

1.3 Keywords

Platelets, erythrocytes, flow cytometry, sorting, human blood plasma, super-resolution microscopy

1.4 Experiment variables

Experimental variables between the samples are only present due to experimental manipulation. Specific subpopulations of particles (erythrocytes and platelets) were sorted based on surface marker expression.

1.5 Experiment design and quality controls

All samples were sorted using single-use disposable high-speed cartridges. Data from the platelet sorting experiment is representative of three independent experiments. Data was not pooled because the starting material was different, measurements were performed on different flow cytometers, and antibody-fluorophore combinations varied. The erythrocyte sorting experiments were performed once. Fluorescence and light scattering calibrations were performed.

1.6 Dates

Samples were collected between June 10th and June 26th, 2024 at the Amsterdam University Medical Center. Flow cytometry data of the experiments on June 26th, 2024 are reported in this study.

1.7 Conclusions

Residual erythrocytes and platelets in plasma were enriched 64x and 40x, respectively. These enriched particles could be imaged using high-resolution dSTORM imaging and fluorescence imaging. Using two complementary techniques, the presence of remaining erythrocytes and platelets in "platelet-free" plasma was confirmed.

2 Sample details

2.1 Sample description

2.1.1 Sample source description

Blood collection was in accordance with the guidelines of the Medical Ethical Committee of the Amsterdam Medical Centre, University of Amsterdam (W19_271#19.421). Blood was collected from a healthy individual. The donor denied having a disease or to use drugs and/or medication.

2.1.2 Sample description

Intravenously collected blood.

2.2 Sample collection

Venous blood was collected using a 21-Gauge needle without tourniquet. The first 2-3 mL of blood was discarded to avoid that activation of endothelial cells and platelets during vein puncture would affect the measurement results. Two tubes of Ethylene Diamine Tetra Acetic acid (EDTA) blood (10 mL; BD Biosciences) were then collected from the donor. Blood was processed within 15 minutes after withdrawal.

2.3 Sample preparation and storage

Plasma preparation

Plasma was prepared by centrifugation of whole blood, according to the guidelines of the American Heart Association. In short, whole blood was centrifuged for 15 minutes at 2,500 x g and 20 °C, with the centrifuge acceleration set at 9, and deceleration set at 1 using a Rotina 380 R centrifuge with a swing-out rotor and a radius of 155 mm (Hettich Zentrifugen, Tuttlingen, Germany). Single centrifuged plasma was collected to exactly 10 mm (using a lego brick) above the buffy coat, and 3.5

mL plasma was then transferred to a 15 mL polypropylene tube (Greiner Bio-One B.V., Alphen aan den Rijn, The Netherlands). The plasma was then centrifuged for 15 minutes at 2,500 x g and 20 °C, with the centrifuge acceleration set at 9, and deceleration set at 1. Double centrifuged plasma was collected to exactly 10 mm above the cell pellet. The concentration of remaining erythrocytes and platelets in double centrifuged plasma was directly assessed by flow cytometry (Cytek Northern Lights). Double centrifuged plasma was used as the input material for sorting experiments on the same day (MACSQuant Tyto Cell Sorter). Remaining plasma was aliquoted into 1.5 mL low-protein binding Eppendorfs (Thermo Fisher Scientific, US) and stored at -80˚C.

2.4 Sample characteristics

Frozen human plasma samples are expected to contain the following particles: erythrocytes, EVs, lipoproteins, platelets, precipitated salt crystals, proteins, and complexes of the aforementioned particles.

2.5 Sample dilution

All samples were diluted 2-fold in DPBS before staining. The staining procedure added an extra dilution factor of 11.25-fold to the overall dilution.

2.6 Sample staining

Supplementary Table 1 shows an overview of the antibodies that were used to stain erythrocytes and platelets in plasma. Prior to staining, the antibodies were diluted in DPBS. For each antibody the optimal dilution factor was determined by titration. To remove aggregates, diluted antibodies were centrifuged at 18,890 g for 5 min at 20 °C. The supernatant minus 10 μL of the starting volume was collected and used for staining.

For erythrocyte detection, each sample was double strained with CD61-PE (phycoerythrin) and CD235a-APC (allophycocyanin). To stain, 1400 μL of 2-fold diluted plasma was incubated with 175 μL of each antibody and kept in the dark for 30 minutes at room temperature. After the incubation, samples were diluted in 15,480 mL DPBS to decrease background fluorescence from unbound reagents.

For platelet detection, each sample was triple strained with CD61-PE, CD235a-APC and CD62p-BV421 (Brilliant Violet 421). To stain, 1000 μL of 2-fold diluted plasma was incubated with 125 μL of each antibody and kept in the dark for 30 minutes at room temperature. After the incubation, samples were diluted in 12,105 mL DPBS to decrease background fluorescence from unbound reagents. CD62p-BV421 results are not shown.

2.7 Fluorescence reagents

**Characteristic measured**	**Analyte**	**Analyte detector**	**Reporter**	**Isotype**	**Clone**	**Concentration (µg mL^{-1})**	**Manufacturer**	**Catalog number**	**Lot number**	**Dilution factor**
Integrin beta 3	CD61	Anti-human CD61 antibody	PE	IgG1 κ	VI-PL2	12	eBioscience	12-0619-42	1999567	4x
Glycophorin A	CD235a	Anti-human CD235a antibody	APC	IgG1	R10	25	R&D systems	FAB12281A	ADGT022 0091	4x
Selectin	CD62p	Anti-human CD62p antibody	BV421	IgG1	AK-4	12.5	BD Biosciences	564038	5295622	8x

Supplementary Table 1. Overview of staining reagents. Characteristics being measured, analyte, analyte detector, reporter, isotype, clone, stock concentration, manufacturer, catalog number and lot number of used staining reagents. The concentration of staining reagents during measurements was 11.1-fold lower than the concentration during staining. APC: allophycocyanin; CD: cluster of differentiation; PE: phycoerythrin, BV: brilliant violet.

3 Flow cytometer

3.1 Model and manufacturer

MACSQuant Tyto, Miltenyi Biotec, Bergisch Gladbach, Germany.

3.1.1 Flow rate and acquisition time

The sorter automatically adjusts the pressure applied on the sample to keep an average transit time of 40 µs. High-speed cartridges were used with a mean flow rate of 8 mL/hour. Cartridges were primed before usage with DPBS. Samples were analyzed until the input chamber was empty. Measurements times per cartridge are mentioned in Supplementary Table 2. The maximum input volume for the High-speed cartridges is 10 mL. To measure more than 10 mL the cartridges were reprimed after measuring 10 mL, and the remaining volume was measured.

Experiment	Measured volume (mL)	Measurement time (min)
Platelets (CD61-PE)	13	94
Erythrocytes (CD235a-APC)	12.5	89

Supplementary Table 2. Measured volumes and measurement times per experiment.

3.1.2 Sample mixing and temperature control

A mixer in the cartridge input chamber ensured the homogeneity of the input sample and was kept at 800 rpm. Temperature control was set to room temperature.

3.1.3 Light sources

The sorter has three lasers, 405-nm, 488-nm, and 640-nm. The three lasers illuminate the microfluidic channel at three independent points to evaluate the transition time of the particle and trigger the sorting valve. The power for all three lasers is 100 mW.

3.1.4 Detectors

Supplementary Table 3 shows an overview of the detectors used in this study.

Detector name	Detected property	Gain (V)	Spectral filter bandwidth (nm)
BSV	Back scattered light violet	610	
BSB	Back scattered light blue	700	
BSR	Back scattered light red	640	
B2	PE fluorescence	480	585/40
R1	APC fluorescence	630	655-730

Supplementary Table 3. Detector name, detected property, gain and spectral filter bandwidth of the detectors used in this study. APC: allophycocyanin; PE: phycoerythrin.

Experiment	Detector	Noise threshold in arbitrary units (a.u.)	Noise threshold in effective scattering cross section (nm^2)(Section 5.3.3)	Noise threshold in optical diameter (nm) (Section 5.3.3)
2x2500 platelets (CD61-PE)	BSV	3.52	112	2426
2x2500 platelets (CD61-PE)	BSB	3.52	45	1661
2x2500 platelets (CD61-PE)	BSR	3.52	104	2872
2x2500 erythrocytes (CD235a-APC)	BSV	2.35	74	2572
2x2500 erythrocytes (CD235a-APC)	BSB	2.35	30	2017
2x2500 erythrocytes (CD235a-APC)	BSR	2.35	69	4131

3.1.5 Trigger detector, noise threshold, and sorting channels

Events to be sorted are delineated by the Tyto software using a noise threshold, a trigger threshold, and a sorting gate. The noise threshold removes background noise events from further analysis and is applied simultaneously on all three backscatter channels (BSV, BSB, BSR). The noise threshold for the three backscattering detectors was set individually for each cartridge. The noise threshold in arbitrary units (a.u.) corresponds to an effective scattering cross section and optical diameter (5.3.3.1), see Supplementary Table 4. The trigger threshold can be used as an additional trigger to filter irrelevant triggered events further. No trigger threshold was set and all events above the noise threshold were considered for sorting. The sorting gate selects particles for sorting into the sorting chamber of the cartridge. The sort gate for erythrocytes sorting was set on BSV versus APC. The sorting gate for platelets sorting was set on BSV versus PE.

Supplementary Table 4. Sample measured, detector used in sort gate, and the noise threshold in arbitrary units, scattering cross section and optical diameter for platelets and erythrocytes (See section 5.3.3.1).

4 Assay controls

We relied on immunofluorescent staining, super-resolution microscopy, transmission electron microscopy, fluorescent microscopy, and calibrated signal flow cytometry intensities to confirm that signals originate from erythrocytes and platelets.

4.1 Unstained controls

Unstained controls were measured with the same dilution factor as the stained plasma samples. Unstained controls were used as a negative reference for setting the fluorescence and scatter gates.

5 Data analyses

To apply calibrations we used customized Rosetta Calibration (v2.05, Exometry, The Netherlands). Sort gates were set manually in the Tyto software before sorting.

5.1 Data sharing

Data are available via: https://doi.org/10.6084/m9.figshare.c.7667636

5.2 Compensation details

No compensation was applied because no fluorophore combinations were used that have overlapping emission spectra.

5.3 Calibrations

5.3.1 Flow rate

The instrument software made automated pressure adjustments to maintain a constant transit time of 40 µs.

5.3.2 Fluorescence calibration

<u>MESF calibration</u>

Calibration of the fluorescence detectors from arbitrary units (a.u.) to molecules of equivalent soluble fluorochrome (MESF) was accomplished using 2-µm APC quantification beads (lot 2364-87, custom-order, Becton Dickinson Biosciences), and 2-µm PE Quantification beads (lot 2364-89, custom-order, Becton Dickinson Biosciences). Supplementary Figure 1 A and B show the 10-base logarithm of the MESF intensities for the MESF beads versus the 10-base logarithm of the measured median fluorescence intensity of each bead population. The data are fitted with a linear function. These fluorescence calibrations were used to assign MESF values to the samples. The data are fitted with a linear function. For each measured plasma sample, we added fluorescent intensities in MESF units to the flow cytometry data files using the following equation:

$\mathrm{I(MESF)} = 10^{a \cdot \log_{10} \mathrm{I(a.u.)} + b}$	Equation 1

where I is the fluorescence intensity, and *a* and *b* are the slope and the intercept of the linear fits in Supplementary Figure 1.

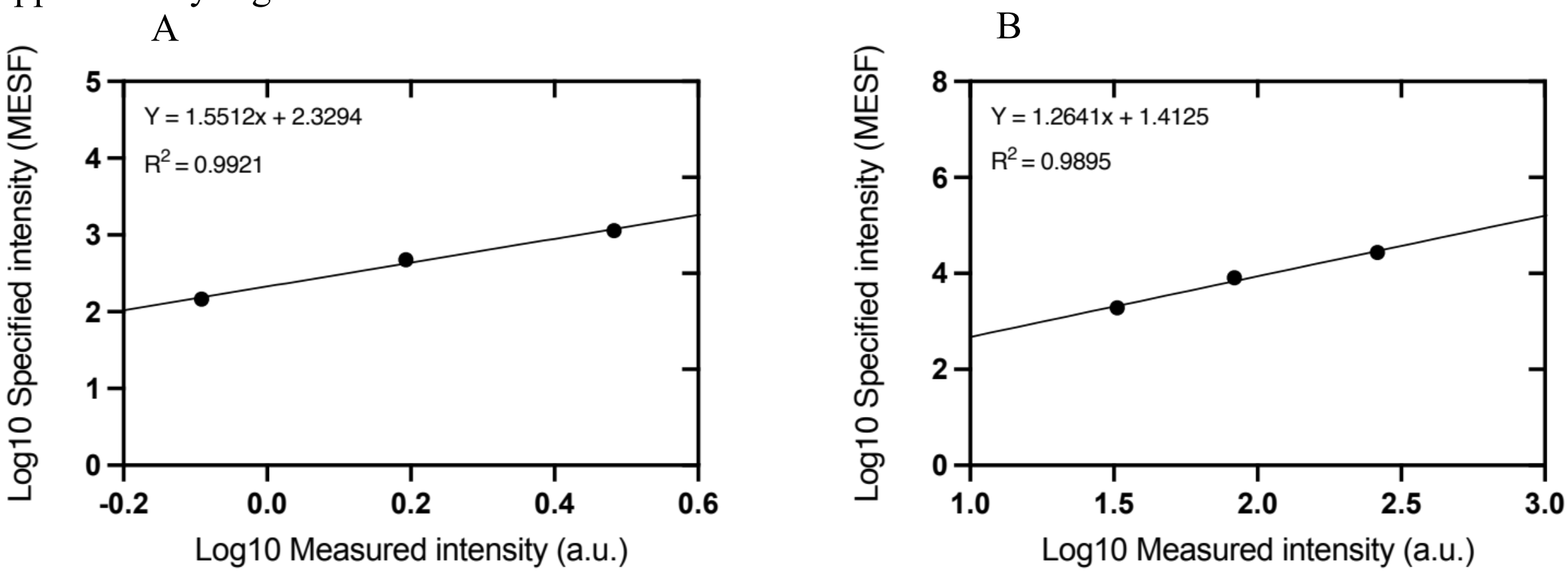

Supplementary Figure 1. Calibration of the fluorescence detectors from arbitrary units (a.u.) to molecules of equivalent soluble fluorochrome (MESF). Logarithmic MESF versus logarithmic median fluorescence intensity for (A) phycoerythrin (PE) and (B) allophycocyanin (APC). Data (symbols) are fitted with a linear function (line).

5.3.3 Light scattering calibration

5.3.3.1 Rosetta Calibration

Rosetta Calibration (v2.05, Exometry, The Netherlands) was used to relate the side scattering intensities measured at a wavelength of 405 nm to the effective scattering cross sections[1] and optical diameter[2] of erythrocytes and platelets. Platelets are modelled as core-shell particles with a core refractive index of 1.38, a shell refractive index of 1.48, and a shell thickness of 6 nm. Because erythrocytes have lost their hemoglobin content they are modelled as core-shell particles with a core refractive index that is lower compared to platelets of 1.35, a shell refractive index of 1.48, and a shell thickness of 6 nm. Supplementary Figure 2 shows a print screen of the light scatter calibrations. The

---

[1] The effective scattering cross section is a hypothetical area of a particle that incoming light must impinge in order to be scattered towards the lens. The calibrated effective scattering cross section axis is independent of refractive index assumptions, but depends on the illumination wavelength and collection angles of the flow cytometer [3].

[2] The optical diameter equals the physical diameter of a particle when (1) the particle is spherical and (2) the particle has the same refractive index distribution as assumed in the model.

scatter calibrations of the Tyto backscatter data are preliminary and only serve as an indication, thorough analysis should be performed to confirm these calibrations.

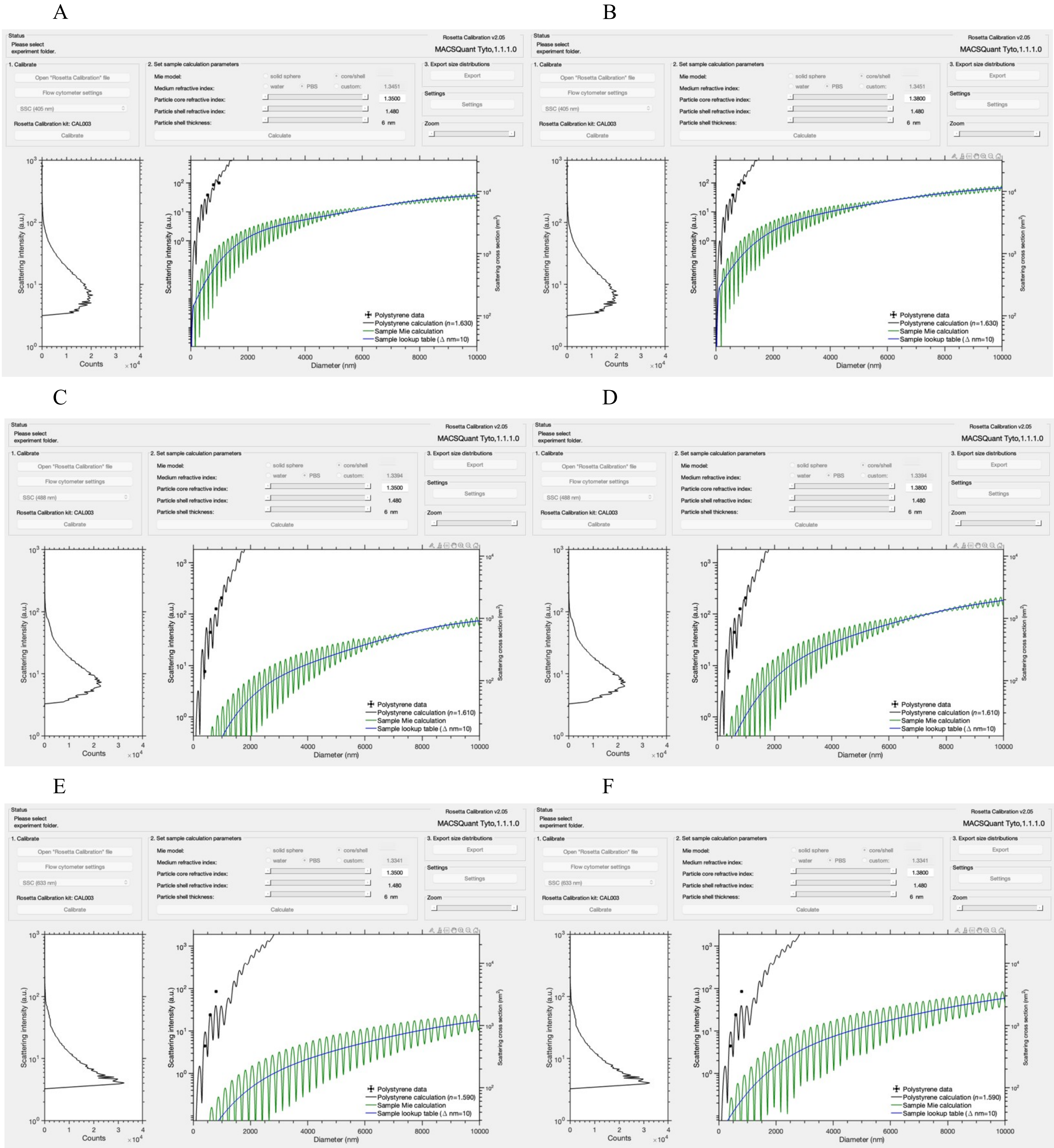

Supplementary Figure 2. Side scattering calibration of the MACSQuant Tyto Cell Sorter (Miltenyi Biotec, Germany) by Rosetta Calibration. To relate scatter to the diameter of erythrocytes are modelled as core-shell particles with a core refractive index of 1.35, a shell refractive index of 1.48, and a shell diameter of 6 nm. Platelets are modelled as core-shell particles with a core refractive index of 1.38, a shell refractive index of 1.48, and a shell diameter of 6 nm. Erythrocyte calibrations are shown in the left column (A, C, E) and platelet calibrations are shown in the right column (B, D, F). Calibration for the violet laser (405 nm) is shown in the top row (A, B), for the blue laser (488 nm) in the middle row (C, D), and for the red laser (633 nm) in the bottom row (E, F).

5.4 Sort gate description and boundaries

The following sort gates have been used

1. Erythrocytes were sorted using a rectangular gate of the APC-H and BSV-H channels. Events were sorted when the APC-H value were between 1.25×10^{1} - 1.25×10^{3} a.u., corresponding to 6.30×10^{3} - 8.95×10^{5} MESF, and the BSV-H values were between 1.6 - 6.3×10^{2} a.u, corresponding to approximately 3.5×10^{2} - 7.7×10^{3} nm.
2. Platelets were sorted using a rectangular gate of the PE-H and BSV-H channels. Events were sorted when the PE-H value were between 1.6 - 2.6×10^{3} a.u., corresponding to MESF, and the BSV-H values were between 2.4 - 1.0×10^{2} a.u, corresponding to approximately 8.0×10^{2} - 3.0×10^{3} nm.

Gates were set based on the 1x2500 platelet and erythrocyte-rich samples.

PDF files with scatter plots of all applied sort gates are available via the online data repository (section 5.1).

6 References

[1] J. A. Lee *et al.*, “MIFlowCyt: the minimum information about a Flow Cytometry Experiment,” *Cytometry A*, vol. 73, no. 10, pp. 926–930, 2008.
[2] B. Bettin *et al.*, “Removal of platelets from blood plasma to improve the quality of extracellular vesicle research,” *J. Thromb. Haemost.*, vol. 20, no. 11, pp. 2679–2685, 2022.
[3] J. A. Welsh *et al.*, “A compendium of single extracellular vesicle flow cytometry,” *J. Extracell. Vesicles*, vol. 12, no. 2, p. e12299, 2023.
