# Supplementary material for: Visual Confirmation of Residual Erythrocytes and Platelets in Human Plasma
Source: Biomark Insights. 2026 Sep 21;21:11772719261481222. doi: 10.1177/11772719261481222 (PMC13728427; doi:10.1177/11772719261481222)
Supplement: Supplementary file 2 [file sj-pdf-2-bmi-10.1177_11772719261481222.pdf]

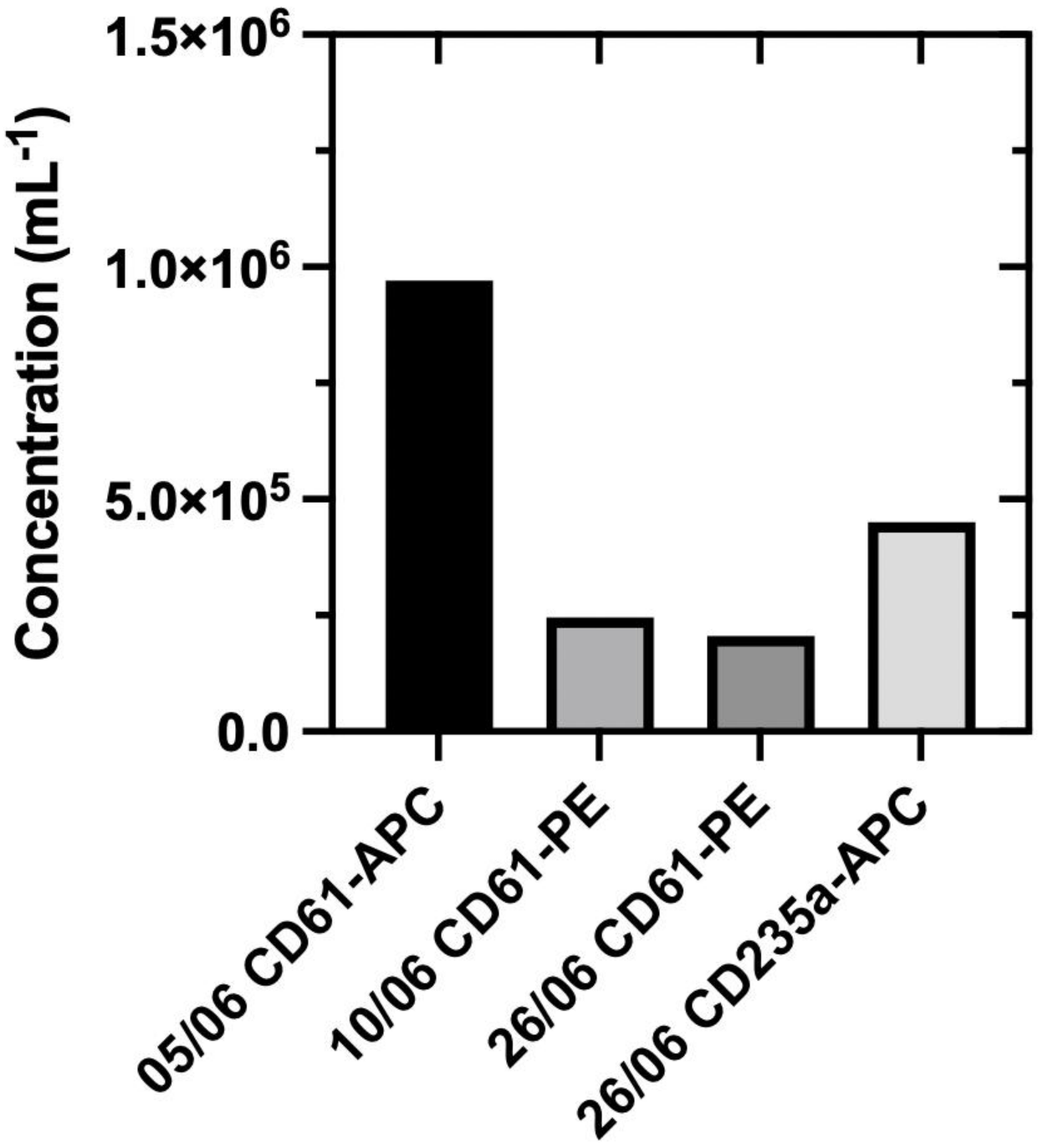

Supplementary Figure 1. Concentration of CD61 and CD235a positive particles per experiment date, measured using flow cytometry. All samples were prepared according to the Methods sections 2.1 and 2.2. Because of different antibody-fluorophore combinations, these data were not combined and have been added to the supplement.
